# Salvage of the Mastectomy Pocket in Infected Implant-Based Breast Reconstruction Using Negative-Pressure Wound Therapy with Instillation and Dwell: A Systematic Review and Meta-Analysis

**DOI:** 10.3390/jcm14082730

**Published:** 2025-04-16

**Authors:** Laura De Pellegrin, Isabel Zucal, Giorgio Treglia, Corrado Parodi, Riccardo Schweizer, Marco De Monti, Yves Harder

**Affiliations:** 1 Department of Plastic, Reconstructive and Aesthetic Surgery EOC, Ospedale Regionale di Lugano, Ente Ospedaliero Cantonale (EOC), 6900 Lugano, Switzerland; laura.depellegrin@gmx.de (L.D.P.); isabel.zucal@eoc.ch (I.Z.); corrado.parodi@eoc.ch (C.P.); marco.demonti@eoc.ch (M.D.M.); 2 Faculty of Biomedical Sciences, Università della Svizzera Italiana, 6900 Lugano, Switzerland; giorgio.treglia@eoc.ch; 3 Division of Medical Education and Research, Ente Ospedaliero Cantonale (EOC), 6500 Bellinzona, Switzerland; 4 Department of Hand Surgery and Plastic Surgery, Luzerner Kantonsspital, 6000 Lucerne, Switzerland; 5 Department of General Surgery, Ospedale Regionale di Mendrisio, Ente Ospedaliero Cantonale (EOC), 6850 Mendrisio, Switzerland; 6 Department of Plastic, Reconstructive and Aesthetic Surgery and Hand Surgery, University Hospital of Lausanne (CHUV),1011 Lausanne, Switzerland; 7 Faculty of Biology and Medicine, University of Lausanne (UNIL), 1015 Lausanne, Switzerland

**Keywords:** negative-pressure wound therapy, NPWTi-d, breast reconstruction, breast implant, periprosthetic breast infection

## Abstract

**Background:** Breast cancer, irrespective of gender, stands as the most prevalent cancer globally, with an annual estimate of 2.3 million new cases. Surgical intervention, including therapeutic mastectomy (excluding prophylactic procedures), is performed on approximately 28% of patients, necessitating subsequent breast reconstruction. Although implant-based breast reconstruction (IBBR) is frequently employed due to its relative ease compared to autologous methods, it presents a notable risk for complications at mid-term such as peri-prosthetic infections. These complications can lead to implant loss and the eventual compromise of the mastectomy pocket. To address these complications, negative pressure wound therapy with instillation and dwell (NPWTi-d) emerges as a promising rescue intervention, known for its capacity to significantly reduce bacterial load and potentially salvage compromised soft tissues. However, the evidence supporting its effectiveness in infected pockets after mastectomy is currently insufficient. This study aims at investigating the efficacy of NPWTi-d in the management of peri-prosthetic mastectomy pocket infection. **Methods:** A thorough literature search has been concluded through PubMed, Web of Science, and Cochrane databases up until 18th March 2025 on evaluating NPWTi-d’s ability to manage peri-prosthetic infections and preserve mastectomy pockets for subsequent reconstruction. Furthermore, a meta-analysis on the salvage rate of the mastectomy pocket was carried out, while for other outcomes, a descriptive analysis was applied. **Results:** Nine studies (n = 230 patients) were included, investigating whether the us NPWTi-d was successful in treating peri-prosthetic infection and preserving the mastectomy pocket for subsequent reconstruction by expander or implant. The pooled salvage rate of the implant-based BR due to the use of NPWTi-d was 86.1% (95%CI: 80.6–91.6%). Preservation of the skin envelope avoided secondary reconstruction after a defined time interval, reducing number and complexity of surgeries and related costs. **Conclusions:** This innovative surgical approach should be considered in selected cases of infected implants after breast reconstruction in breast cancer centers. However, the actual low level of evidence is based on case series, and it is not possible to define generally accepted recommendations for the use of NPWTi-d to save the mastectomy pocket.

## 1. Introduction

Breast cancer has recently overtaken lung cancer in terms of incidence, and independent of gender, it has become the cancer with the highest incidence overall, with an estimated 2.3 million new cases each year [1], Excluding all prophylactic mastectomies performed in gene carriers and high-risk patients, the US Surveillance, Epidemiology, and End Results program reports a mastectomy rate of about 28% [2]. In light of a high survival rate in the early breast cancer stages undergoing stage-adapted multi-modal therapy, this translates into a large pool of patients that require organ or breast reconstruction [3,4]. Nipple- or skin-sparing mastectomy is an oncologically safe surgical therapy compared to modified radical mastectomy and is, therefore, accepted as a suitable approach for a large spectrum of local breast cancer stages, allowing preservation of the breast’s skin envelope [5]. Acknowledging that patients may opt not to pursue reconstruction due to personal preferences or other health concerns, this treatment modality entails an approximate rate of 70% immediate implant-based breast reconstructions (IBBR) [6,7]. Overall, IBBR is the most common reconstructive procedure performed worldwide after mastectomy, both for oncologic and prophylactic indications. From a technical aspect, IBBR is less complex than flap-based breast reconstruction (BR); moreover, it is less time-consuming and resource-dependent [8].

Despite these advantages of IBBR over autologous BR, as well as explicit patient preference for implant-based reconstruction for various personal reasons, there are some important drawbacks, including a rather high rate of mid- to long-term complication other than infection, such as capsular contraction and implant rotation and/or displacement, as well as implant rupture. Despite the fact that it is technically less demanding as well as more frequently available when compared to autologous BR, IBBR almost never offers a long-lasting or definitive reconstructive solution [9,10]. The latter can result in a loss of both implant and its pocket. While salvage attempts are readily made in cases of mild infection, terminology defined by Kanapathy et al. [11] as “defined as warmth, swelling and/or cellulitis that was responsive to antibiotic therapy”, cases of severe peri-prosthetic infection are typically considered unsuitable for salvage [9]. According to Spear’s classification, severe breast infection is defined as follows: generalized erythema, purulent drainage ± systemic signs of infection, threatened implant exposure with mild infection, and threatened devices with concurrent severe infection, as well as actual device exposure with significant or severe infection [9].

Implant removal and creation of a prolonged flat-chest situation of approximately 3 months, followed by secondary reconstruction after months or years, if desired by the patient, has traditionally been the standard in the treatment of severe peri-prosthetic infections [12]. This approach is currently considered the safest way to definitively treat implant-associated infections. Although successful in overcoming the infection, this accepted stepwise approach has been associated with unfavorable outcomes, such as excessive scarring and a high rate of symptomatic capsular contracture, resulting in breast distortion and, therefore, an unpleasant aesthetic outcome [12]. Additionally, salvage of the skin pocket of infected or exposed implants with explantation and one-stage free flap replacement, as described by Harris et al., is another option that must be discussed with the patient [13]. Ultimately, these situations significantly increase psychological distress by delaying the reconstruction of the patient’s integrity and prolonging the flat-chest situation [14,15,16].

Negative-pressure wound therapy with instillation and dwell (NPWTi-d) is an established treatment used to treat a wide range of wounds, be them acute, chronic, closed, open, infected, or non-infected wounds, as well as any combination thereof [17]. NPWTi-d provides additional benefits to the known effects of standard NPWT without instillation and dwell. Continuous wound cleansing reduces local bacterial load, resulting in increased local blood flow, reduced tissue edema, as well as development of granulation tissue and enhanced tissue healing [16,17,18]. Gabriel et al. demonstrated that NPWTi-d could significantly reduce the mean time span until bioburden was decreased and wound closure was achieved, compared with traditional wet-to-moist wound care [18]. Accordingly, it seemed obvious to explore if NPWTi-d could be an option to preserve the mastectomy pocket in peri-prosthetic infections after IBBR, as first described by Meyodi et al. in 2015 [19]. Although several studies describe the use of NPWTi-d to treat infected mastectomy pocket, there is a lack of evidence regarding its efficacy and salvage rate in the setting of peri-prosthetic breast infection.

The aim of this study was to compare the various outcomes of NPWTi-d in the management of peri-prosthetic breast infection, in particular salvage of the mastectomy pocket.

## 2. Materials and Methods

### 2.1. Literature Search

A review protocol was developed based on the preferred reporting items for systematic reviews and meta-analyses (PRISMA) statement [20]. A comprehensive literature search was performed by two independent authors (L.DP., M.DM.) in the literature databases PubMed, Web of Science, and the Cochrane Library up to 18th March 2025. The following research terms were used: “NPWTi-d AND breast reconstruction” or “NPWTi-d AND breast prosthesis” or “NPWTi-d AND breast implant” or “NPWT AND breast reconstruction” or “NPWT AND breast prosthesis” or “NPWT AND breast implant” or “negative pressure wound therapy with instillation AND breast reconstruction” or “negative pressure wound therapy with instillation and breast prosthesis” or “negative pressure wound therapy with instillation AND breast implant”. Randomized controlled trials, case-control studies, and prospective and retrospective cohort studies, as well as case series with at least five patients were included. There were no limitations regarding the follow-up period. Studies that did not include peri-prosthetic breast infection in patients who previously underwent mastectomy treated with either NPWTi-d or NPWT were excluded. Pre-clinical studies, ex-vivo studies, literature studies, and studies in languages other than English were also excluded.

### 2.2. Data Extraction

**Figure 1:**
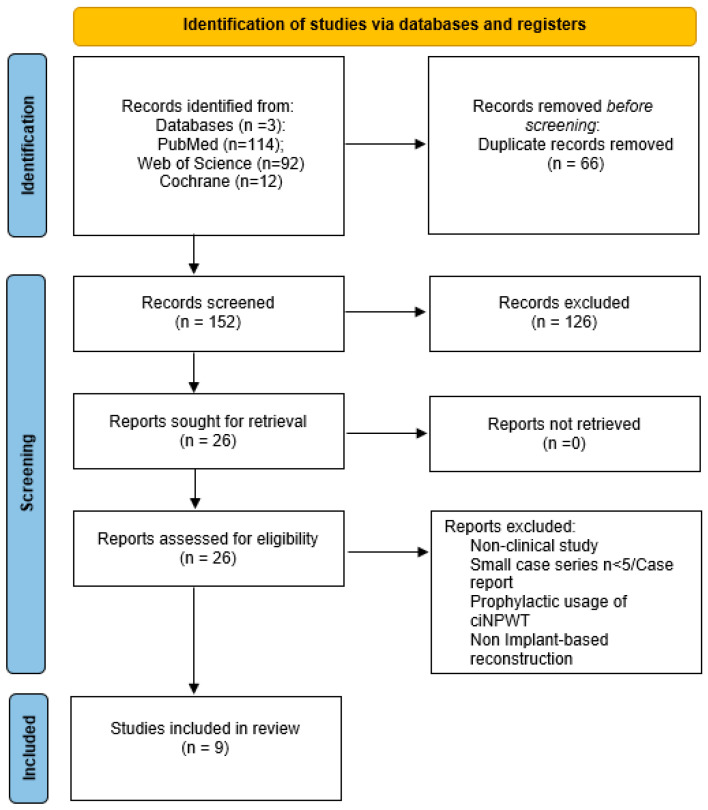
Review protocol according to PRISMA guidelines of included studies.

Two independent reviewers, L.DP. and M.DM., screened all the titles and abstracts. After this initial screening, the articles that met the inclusion criteria were analyzed for full-text eligibility and excluded if they met any one of the exclusion criteria (Figure 1). In case of disagreement between the two reviewers, a third reviewer (Y.H.) was consulted to reach consensus.

A digital table was created for data extraction prior to the study using Excel (Microsoft Excel. Version 2016). The following data were extracted from each included study: country, number of surgical procedures, number, gender and age of the participants, duration of follow-up, wound characteristics, NPWTi-d therapeutic settings, infection rate, time until final wound closure, complication rate, total duration of treatment, and length of hospital stay. Following this independent data collection, the reviewers compared the extracted data.

### 2.3. Assessment of Risk of Bias and Quality of Evidence of the Included Studies

The Downs and Black’s “Checklist for Measuring Quality” was used to evaluate the risk of bias [21]. Risk assessment for bias and quality of evidence were completed independently for all outcomes by two authors (L.DP., M.DM.), and a third author (Y.H.) resolved any discrepancies in reaching consensus.

### 2.4. Statistical Analysis—Quantitative Synthesis

The outcome measure for the meta-analysis (quantitative synthesis) was the rate of mastectomy pocket salvage due to the surgical intervention using NPWTi-d. First, data on the number of breasts treated and mastectomy pocket salvage were extracted from each article and included in the meta-analysis to obtain the rate of mastectomy pocket salvage on a per-breast analysis. Then, the pooled rate was calculated through a proportional meta-analysis. A random effect model according to DerSimonian and Laird was used for the pooled analysis [22]. Pooled data were provided along with 95% confidence interval values (95% CI), and the results of the meta-analysis were graphically displayed using a forest plot. The I-square (I2) or inconsistency index was calculated to evaluate the statistical heterogeneity, and values > 50% were considered as a marker of significant statistical heterogeneity. Subgroup analyses were planned only in case of significant statistical heterogeneity. Publication bias was assessed with the use of the Egger’s test. OpenMeta (analyst)^®^ software v0.24.1 (Rockville, MD, USA) was used for the meta-analysis.

## 3. Results

### 3.1. Literature Search Results

A total of 221 publications were identified according to the predefined inclusion criteria. After removal of all duplicates, 152 titles and abstracts remained to be screened, resulting in 26 articles for full-text review. Seventeen of these did not meet the inclusion criteria. Accordingly, nine studies (four from the USA, three from Australia, and one from Germany and UK each) have finally been included in this review (Figure 1). Details of the included studies are summarized in Table 1, whereas details regarding both the IBBR and the salvage protocol are summarized in Table 2.

### 3.2. Details of the Included Studies

A total of 230 patients were included in this review; of them, 167 patients were treated with NPWTi-d and compared to 63 patients treated with the standard-of-care in case of infected mastectomy pocket, consisting of either autologous or two-staged implant-based BR, meaning that further skin expansion with an expander was necessary or delayed direct implant reinsertion without pre-expansion, terminology used by Haque et al., indicating implant reinsertion without secondary expansion beforehand [26]. Follow-up time reported for each study varied significantly, ranging from 3 to 39 months (mean: 16.0 months).

### 3.3. Therapeutic Settings

The mean length of NPWTi-d therapy was 4.6 days (range 55 h–8.5 d), resulting in a mean hospital stay of 9.1 days (range 3 d–12 d). Different instillation solutions were used, the most common one being saline (n = 5/9 studies). Other solutions were 0.1% polyhexanide and 0.1% betaine (Prontosan^®^, B. Braun Medical AG, Sempach, Switzerland), Polyhexanid 0.4 mg/mL (Lavasept^®^, B. Braun Medical AG, Sempach, Switzerland) antiseptic solution, and acetic acid.

### 3.4. Quantitative Analysis

**Figure 2:**
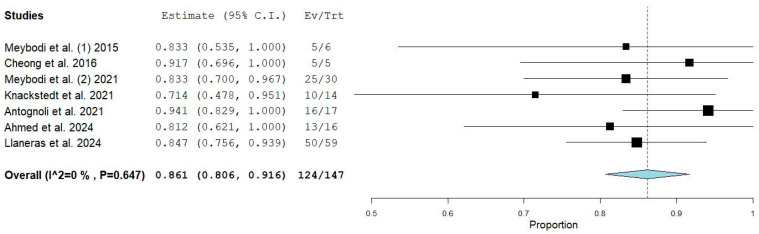
Meta-analysis of mastectomy pocket salvage rate [19,23,24,27,28,29,30].

A total of seven studies, encompassing 147 breasts, were eligible for inclusion in the meta-analysis based on their methodological design. The pooled salvage rate of implant-based breast reconstruction (BR) associated with the use of negative pressure wound therapy with instillation and dwell time (NPWTi-d) was 86.1% (95% CI: 80.6–91.6%) (Figure 2). Figure 2 presents a forest plot illustrating the results of our meta-analysis. Each included study is depicted by a black square, the size of which reflects its relative weighting within the analysis. The horizontal black lines extending from the squares represent the respective 95% confidence intervals. A vertical dashed red line indicates the overall pooled estimate, while a blue diamond at the bottom of the plot represents the combined proportion along with its confidence interval. 

The I^2^ test was 0%, demonstrating the absence of significant statistical heterogeneity among the included studies. Sub-group analyses have not been carried out, considering the value of the I^2^ test. The Egger’s test (*p* = 0.3) demonstrated the absence of any significant publication bias.

### 3.5. Mastectomy Pocket Volume Preservation and Surgical Technique

A major benefit within the usage of NPWTi-d is the concurrent preservation of the skin envelope as well as the mastectomy pocket itself, whereas the current standard treatment approach without implant and pocket salvage provides delayed re-implantation, often after several months [26]. By contrast, Cheong et al. [24] reported re-insertion of breast implants after 7 days after NPWTi-d treatment, with successful preservation of the breast cavity volume, allowing re-insertion of implants of the same size, as previously removed. Furthermore, the surgical techniques vary or are not described in detail, consisting of a combination of oral/intravenous antibiotics and operative debridement with varying degrees of capsulectomy, as well as pocket irrigation, as shown in Table 2.

### 3.6. Change in Bioburden and Clinical Infection

Gruener et al. investigated the outcomes in their cohort of 13 patients, focusing on changes in inflammatory parameters and bioburden with the usage of NPWTi-d [25]. Thereby, NPWTi-d could demonstrate a significant reduction of C-reactive protein (55.3 mL/L prior NPWTi-d and 15.5 mL/L at wound closure, *p* = 0.0002), as well as for leukocyte count (8.16 × 10^3^/µL prior NPWTi-d and 5.83 × 10^3^/µL at wound closure *p* = 0.0002). The authors could also show a significant lower bioburden at the end of NPWTi-d treatment (*p* = 0.002), as well as a significantly reduced number of involved bacterial stems identified on microbiological examinations before and after the treatment (*p* = 0.001). This was assessed by determining the extent of bacterial colonization of the implant pocket on an ordinal scale. Due to the heterogeneity of the bacterial colonization, the total amount of all bacteria was calculated by summing these ordinal-scaled numbers of each bacterium.

### 3.7. Time Interval Between Implantation and Infection

Six studies reported the interval between implant insertion and the occurrence of infection, with an average time of 110 days (range 33 days–36 weeks). This is mentioned to emphasize the wide range of infection onset, with some cases appearing immediately after surgery, while others develop later after complete wound healing, possibly due to contaminated peri-prosthetic tissue or hematogenous spread.

### 3.8. Predictability of Mastectomy Pocket Salvage

Llaneras et al. remain the sole researchers investigating potential predictive risk factors for complications following NPWTi-d intervention [30]. In the present analysis, 27% of the cases experienced post-discharge complications within a three-month follow-up period, including major infection (n = 8), wound dehiscence (n = 5), minor infection (n = 1), and seroma (n = 1). These adverse events resulted in explantation and reconstructive failure in 9 cases (15%), while 50 cases (85%) achieved successful salvage. Univariate logistic regression analysis identified no significant associations between successful salvage and established risk factors, except for implant exposure at initial presentation, which was associated with a significantly lower likelihood of salvage success (odds ratio [OR] = 0.20, *p* = 0.03). In the final multivariate model, implant exposure remained a significant predictor of unsuccessful salvage (OR = 0.19, *p* = 0.04), while prolonged outpatient antibiotic therapy demonstrated borderline significance (OR = 0.89 per additional day, *p* = 0.11).

## 4. Discussion

Through this systematic review, the potential benefit of NPWTi-d in managing severe peri-prosthetic infections following implant-based breast reconstruction (IBBR) is shown to contribute to salvaging the infected mastectomy pocket. This highlights the significant role of negative-pressure wound therapy with instillation and dwell in the treatment process.

An integral aspect of the NPWTi-d treatment, as highlighted by all authors, involves repeated intraoperative adjustments of the NPWTi-d system every 48–72 h, accompanied by repeated microbiological assessments, as recommended by current guidelines [31]. Meybodi et al. executed an average of 3.7 takebacks to the operating room (OR) [28]. Additionally, the same author reported an average of 2.3 OR sessions in their 2017 paper, achieving in both studies a salvage rate of 83% [19]. Cheong et al. in turn, reported a salvage rate of 100% (n = 5/5) with more than two takebacks to the OR [24]. Since some authors report the number of NPWT changes performed in the operating room without specifying the intervals between changes, and others only provide the overall “treatment duration” without mentioning the number of NPWT changes, calculating an overall mean surgery time does not yield accurate or representative results.

Antognoli et al. described his approach consisting of implant removal, surgical debridement, extensive wound cleansing, and single application of NPWTi-d for less than 5 days, administrating intravenous antibiotic therapy on an in-patient basis [23]. This specific protocol has the advantage of a rather rapid postoperative course, resulting in a mastectomy pocket salvage rate of 94%, facilitating an early resumption of adjuvant therapy if needed, as well as return to normal life, eventually providing significant emotional benefits to patients. Antognoli’s protocol notably contrasts with other protocols that necessitate multiple returns to the operating room for surgical debridement and replacement of the NPWTi-d device to achieve a reported salvage rate of 94% (n = 15/16) [23]. A key point of interest regarding the initial debridement is the absence of consistent definitions or clear guidelines for the extent of capsulectomy during the salvage process. Additionally, there are no established criteria for determining successful pocket salvage and subsequent implant reinsertion. Meybodi et al. stand as the sole source providing a supplementary digital content entitled ‘Protocol of Implant Salvage in Infected Cases’, which we wish to reference [28].

Although Gruener et al. reported a reduction in inflammatory markers, we do not define pocket salvage success based on a single parameter [25]. In our clinical practice, we follow a detailed in-house protocol for the use of NPWTi-d in salvaging a mastectomy pocket compromised by peri-prosthetic implant infection. Our protocol involves the removal of the implant and extensive surgical debridement, ideally incorporating complete capsulectomy. Multiple intraoperative biopsies are obtained from all four breast quadrants both before and after thorough debridement and irrigation with normal saline (NaCl). To ensure precise microbiological and histopathological evaluation, we favor individual tissue samples over swabs.

To minimize contamination risk, the surgical team replaces gloves and instruments prior to NPWT device insertion. The NPWT sponge is placed around either a sizer or implant to act as a spacer and prevent excessive retraction of the skin envelope. Following the manufacturer’s guidelines, the instillation and dwell system is replaced every 72 h [31]. During each intraoperative return, biopsies from all four quadrants are repeated to monitor for microbiological colonization or pocket sterility. Once negative cultures are confirmed (typically after five days), the new implant is reinserted. However, the decision to reinsert the implant is based on a comprehensive assessment, including clinical judgment and intraoperative findings, rather than solely on inflammatory markers, which may be influenced by the surgery and the open-wound treatment with NPWT.

The second variable associated with these protocols of instillation is the choice of the topical solution. The majority uses saline, though, the use of 0.1% polyhexanide and 0.1% betaine (Prontosan^®^), Polyhexanid 0.4 mg/mL (Lavasept^®^) antiseptic solution and acetic acid has also been described. In line with findings in orthoplastic surgery, NPWTi-d using a topical solution—be it antiseptic or not—shows a more effective reduction in the number of pathogenic species within a wound compared to NPWT without instillation and dwell [32]. Gruener et al. were the first to confirm this fact that was already known from orthoplastic surgery. Similarly, they could show in peri-prosthetic breast infection, after mastectomy, a significantly lower bioburden and a reduced number of bacterial stems found on microbiological examinations, as well as a significant reduction of the principal inflammatory parameters [25].

Considering the merits and drawbacks of topical solutions, saline stands out for its availability, low cost, and low cytotoxicity, making it an attractive choice for NPWTi-d. However, solutions containing surfactants are favored due to their capacity of reducing the presence of biofilms more efficiently, while supporting a friendly environment for a subsequent use of a breast implant. Given limited data currently available and the wide range of solutions used for instillation, future studies could explore the effects on bacterial load and presence of biofilm, depending on the use of different types of cleansing solutions, the duration of administration, and take-backs in OR to change the advanced dressing [23].

An important aspect to consider is the cost–benefit analysis. Patients undergoing NPWTi-d salvage demonstrate cost savings in the long run compared to those undergoing implant removal and delayed reconstruction in cases with infection. This is attributed to the fact that, in cases of IBBR, due to the scarred and retracted skin caused by tissue contraction and scarring, this frequently requires the use of a new tissue expander and subsequent re-expansion to create a new implant pocket or the need for more complex flap-based BR. This results in a longer reconstructive journey with more outpatient visits for tissue expansion and surgical planning and prolonged absenteeism [14,27]. Accordingly, Antognoli’s group reported potential total savings of USD 58,275 using the single application NPWTi-d protocol compared to patients undergoing a temporary flat-chest situation, followed by secondary BR using implants or flaps [23].

All studies that were considered in this study report the use of systemic antibiotic therapy that is usually started empirically and then adapted according to findings in cultures resulting from tissue biopsies and sonicated implants and systemic toxicity. The duration of the antibiotic treatment varies among studies. Meybodi et al. [19,28] administered iv and oral antibiotics for a time-period of 2–4 weeks after surgery, whereas Accurso et al. stopped its administration after removal of suction drains [33]. Of interest, Knackstedt et al. report a total of 25% negative microbiological cultures resulting from wound swabs in their study including 12 patients. Accordingly, they proposed a generic antibiotic treatment with cefazolin, without specifying the treatment protocol [27].

Since multimodal therapy of early breast cancer results in excellent survival rates nowadays, the aesthetic aspects increasingly gained importance and as has the quality of the salvaged mastectomy pocket. The reported incidence of capsular contracture after breast reconstruction in general varies between 2% and 30% [34,35]. This rate is reported as high as 33% after breast implant-related infection [36]. Only Antognoli et al. reported about two cases (12.5%) developing this complication after salvage of the infected mastectomy pocket in IBBR, of which one underwent adjuvant radiotherapy [23].

Patient-reported outcomes (PROMs) are crucial in assessing the success of interventions. Haque et al. evaluated a total of 14 patients in the NPWTi-d group as well as 13 of the control group by using the BREAST-Q questionnaire, reporting higher patient satisfaction with the breast (*p* = 0.032) and a higher satisfaction with the implant (*p* = 0.061), when it comes to the NPWTi-d group [26].

In this systematic review, we identified that the only other review addressing this topic, published in November 2024, aligns with our interpretation of the current literature [16]. Both reviews analyze the same studies, reporting similar findings regarding the efficacy of NPWTi-d in peri-prosthetic breast pocket salvage. However, this current review incorporates additional studies due to a more comprehensive and current literature search, reflecting the most up-to-date evidence available. In addition, this work extends beyond the existing review by including a meta-analysis specifically focused on mastectomy pocket salvage rates, providing a quantitative synthesis of the available data.

The literature search Initially identified 152 potential articles, yet only 9 met the inclusion criteria, highlighting the scarcity of high-quality evidence in this field. The available studies are predominantly retrospective analyses or prospective case series, with a notable lack of randomized controlled trials. Consequently, the meta-analysis is limited by the relatively small number of studies, restricting the extent to which findings could be pooled. Furthermore, only two studies included direct comparisons between intervention and control groups, underscoring the need for more robust comparative evidence. Nevertheless, no publication biases were detected, partly due to our methodological approach of excluding small case series with five or fewer cases; to enhance the reliability of our results. By addressing these knowledge gaps, this review and meta-analysis contribute valuable insights into the current understanding of NPWTi-d for peri-prosthetic infections and mastectomy pocket salvage, while also emphasizing the need for further well-designed clinical trials.

## 5. Conclusions

This analysis of existing literature supports an approximately 86% success rate in salvaging mastectomy pockets using NPWTi-d for infected IBBR. This highlights its potential as a treatment option in select cases of peri-prosthetic infection. However, the optimal timing and type of instillation remain uncertain, underlining the fact for further research, particularly in the absence of clear international recommendations or guidelines. Therefore, future studies are essential to confirm the efficacy of this technique and establish standardized indications.

## Figures and Tables

**Table 1:** Included studies.

Author	Publication Year	Country	Study Design	N° of Patients	N° of Breasts	Instillation Solution	Mastectomy Pocket Salvage Rate (n/Breasts)
Antognoli et al. [23]	2021	USA	Retrospective comparative	NPWTid (n = 16) vs. standard of care (n = 9)	NPWTid = 17 vs. non NPWTid = 9	0.1% polyhexanide + 0.1% betaine (Prontosan)	16/17
Cheong et al. [24]	2016	Australia	Case series	NPWTid n = 5	5	Saline	5/5
Gruener et al. [25]	2022	Germany	Retrospective non-comparative	NPWTid n = 12	13	Polyhexanid 0.4 mg/mL (Lavasept)	n.o.
Haque et al. [26]	2021	UK	Retrospective comparative	NPWTid (n = 20) vs. standard of care (n = 20)	NPWTid = 20 vs. non NPWTid = 20	Saline	n.o.
Knackstedt et al. [27]	2021	USA	Case series	NPWTid n = 12	14	n.r.	10/14
Meybodi et al. [28]	2021	Australia	Case series	NPWTid n = 28	30	Saline, Acetic acid, 0.1% polyhexanide + 0.1% betaine (Prontosan)	25/30
Meybodi et al. [19]	2015	Australia	Case series	NPWTid n = 5	6	Saline (changed accordingly to germs to Acetic acid or Prontosan)	5/6
Ahmed et al. [29]	2024	USA	Retrospective comparative	NPWTid (n = 13) vs. standard of care (n = 34)	NPWTid = 16 vs. non NPWTid = 60	Oxychlorosene	13/16
Llaneras et al. [30]	2024	USA	Multicentric retrospective cohort	NPWTid n = 56	59	Saline, Prontosan and oxychlorosene	50/56

NPWTid, negative-pressure wound therapy with instillation and dwell time; n.r., not reported; n.o., not observed.

**Table 2:** IBBR details and salvage protocol.

	IBBR Details	Presence of Allogenic Material for Implant Coverage	Previous Radiotherapy	Surgical Treatment in Addition to Implant Removal	Implant/Expander	Type of Surgery in Case of Failure
Meybodi et al. [19]	Sub-pectoral placement (n = 6/6, 100%)	Biologic † (n = 1/6, 17%) Synthetic * (n = 2/6, 33%) None (n = 3/6, 50%)	n = 1/6 (17%)	Debridement	3/6 (50%) infected tissue expanders → 3/6 expander insertion instead (1 failure) 3/6 (50%) infected implants → 3/6 expander insertion instead	Bilateral autologous breast reconstruction
Antognoli et al. [23]	Sub-pectoral placement (n = 13/16, 81%) Pre-pectoral placement (n = 3/16, 19%)	NR	n = 0/12	Debridement	9/16 (56%) infected tissue expanders → 3/9 implant insertion instead, 6/9 expander insertion instead 7/16(44%) infected implants → 3/7 expander insertion instead, 4/7 implant insertion instead (1 failure)	1 flat chest for 5 months followed by delayed breast reconstruction with tissue expansion and subsequent exchange for implant
Haque et al. [26]	NR	NR	NR	Debridement	20/20 infected implants → 20/20 new implant insertion instead	NR
Cheong et al. [24]	Sub-pectoral placement (n = 2/5 expanders, 40%) NR for implants	NR	n = 2/5 (40%)	NR	3/5 (60%) infected implants, 2/5 (40%) infected tissue expanders → 5/5 implant insertion instead	NR
Gruener et al. [25]	NR	NR	n = 4/13 (31%)	Total capsulectomy	NR	NR
Meybodi et al. [28]	Sub-pectoral placement (n = 25/30, 83%)	Synthetic * (n = 17, 57%) Biologic † (n = 6, 20%) Autologous ‡ (n = 5, 17%) None (n = 2, 6%)	n = 2/30 (7%)	Debridement	16/30 (53%) infected tissue expanders, 14/30 infected implants (47%) → 24/30 (80%) tissue expander, 5/30 (13%) implant and 1/30 (3%) expansion without replacement due to concerns about delay of AC instead	NR
Knackstedt et al. [27]	NR	NR	n = 2/12 (17%)	NR	12/12 infected implants → 10/12 implant insertion instead	Autologous reconstruction in 12 cases, of which 1 chose for autologous reconstruction following second IBBR failure
Ahmed et al. [29]	NPWTid: Pre-pectoral n = 12 (92.3%), Subpectoral n = 1 (7.7%) Non NPWTid: Pre-pectoral n = 32 (94.1%), Subpectoral n = 2 (5.9%)	Acellular dermal matrix or mesh NPWTid n = 12 (92.3%), non NPWTid n = 34 (100%)	NPWTid n = 0 vs. non NPWTid n = 2 (5.9%)	Capsulectomy and complete removal of mesh if present	NPWTid: TE to TE 8/16 (50%), TE to implant 2/16 (12.5%), Implant to implant 6/16 (37.5%)	Autologous reconstruction or delayed implant reinsertion
Llaneras et al. [30]	Pre-pectoral n = 44 (78.6%)	Mesh/ADM n = 51 (91.1%)	n= 3 (5.4%)	Debridement	TE in 65% (n = 38); Implants 36% (n = 21)	NR

IBBR: implant-based breast reconstruction; NR = not reported; * Synthetic mesh included TiLOOP bra, TiLOOP bra pocket, SERI surgical scaffold, and TIGR matrix; † biologic mesh included the following acellular dermal Matrices-Veritas, Strattice, FlexHD, and Biodesign; ‡ autologous included lipodermal flap, AC adjuvant chemotherapy, TE = tissue expander.

## Data Availability

No new data were created or analyzed in this study, therefor data sharing is not applicable to this article.

## Abbreviations

The following abbreviations are used in this manuscript:

NPWTi-d	Negative pressure wound therapy with instillation and dwell
IBBR	Implant-based breast reconstruction
TE	Tissue Expander
